# Towards Full Citizenship: Correlates of Engagement with the Gender Identity Law among Transwomen in Argentina

**DOI:** 10.1371/journal.pone.0105402

**Published:** 2014-08-18

**Authors:** María Eugenia Socías, Brandon D. L. Marshall, Inés Arístegui, Virginia Zalazar, Marcela Romero, Omar Sued, Thomas Kerr

**Affiliations:** 1 Fundación Huésped, Buenos Aires, Argentina; 2 Department of Epidemiology, Brown University School of Public Health, Providence, Rhode Island, United States of America; 3 Association of Transvestites, Transsexuals, and Transgenders of Argentina (A.T.T.T.A.), Buenos Aires, Argentina; 4 British Columbia Centre for Excellence in HIV/AIDS, St. Paul's Hospital, Vancouver, BC, Canada; 5 Department of Medicine, University of British Columbia, St. Paul's Hospital, Vancouver, BC, Canada; UCSF, United States of America

## Abstract

**Introduction:**

In May 2012, Argentina passed its “Gender Identity” Law, which aimed to address the legal invisibility, discrimination and marginalization that transgender individuals have historically faced. The aim of this study was to explore factors associated with engagement with the Gender Identity Law among transwomen living in Argentina.

**Methods:**

Data were derived from a 2013 nationwide, cross-sectional study involving transwomen in Argentina. Using multivariate logistic regression, we assessed the prevalence and factors associated with acquiring a gender-congruent identity card within the first 18 months of enactment of the Gender Identity Law.

**Results:**

Among 452 transwomen, 260 (57.5%) reported that they had obtained a new gender-congruent identity card. In multivariate analysis, factors positively associated with acquiring a new ID were: previously experiencing discrimination by healthcare workers (adjusted odd ratio [aOR] = 2.01, 95% CI: 1.27–3.20); having engaged in transition procedures (aOR = 3.06, 95% CI: 1.58–5.93); and having a job other than sex work (aOR = 1.81, 95% CI: 1.06–3.10). Foreign born transwomen were less likely to have obtained a new ID (aOR = 0.14, 95% CI: 0.06–0.33).

**Conclusions:**

More than half of transwomen in our sample acquired a new gender-congruent ID within the first 18 months of enactment of the Gender Identity Law. However, access to and uptake of this right has been heterogeneous. In particular, our findings suggest that the most empowered transwomen may have been among the first to take advantage of this right. Although educational level, housing conditions, HIV status and sex work were not associated with the outcome, foreign-born status was a strong negative correlate of new ID acquisition. Therefore, additional efforts should be made in order to ensure that benefits of this founding policy reach all transwomen in Argentina.

## Introduction

*Transgender* is an umbrella term that encompasses the diverse population of individuals whose gender identities and/or gender expressions differ from the sex assigned to them at birth [1], [2]. Throughout the globe, transgender individuals suffer discrimination and social stigma, low access to education, and limited employment and housing opportunities [3]–[7]. Marginalization and unmet basic needs may help explain why transgender individuals often engage in high-risk activities (e.g., sex work), and why this population suffers from a disproportionate burden of HIV infection and other health problems [8]–[11]. Unable to obtain jobs due to low access to education, barriers to legal recognition, and/or discrimination, many transwomen turn to sex work to meet their basic needs [12]–[14]. This social and economic marginalization and high burden of health problems mutually reinforce one another, increasing the many vulnerabilities observed within this population [1], [15], [16].

Public health research has shown the importance of discriminatory sociocultural environments and public policies in shaping health outcomes among transgender persons and other sexual minorities [5], [17]. For example, data from the United States indicate that sexual minority persons living in states with same-sex marriage bans were more likely to be diagnosed with psychiatric disorders, whereas living in states where same-sex relationship are legally recognized was associated with fewer mental health care visits [18], [19]. Argentina has been leading significant regional and worldwide efforts to improve equity for LGBT persons, and to address the unique needs of these vulnerable populations, such as through the legalization of same-sex marriage (including full adoption rights) in 2010 [20].

Despite these reforms, transgender individuals in Argentina have continued to suffer exclusion from full citizenship. This limited access to legal and civil rights, as well as social programs (e.g., housing assistance) has, in turn, largely contributed to frequent discrimination in all spheres of life and continued high levels of vulnerability for transwomen [5], [21], [22]. In response to this and other related public health and social challenges, Argentina passed a progressive “Gender Identity Law” (Law 26,743 24/05/2012) in May 2012 [23]. This Law establishes a framework based on equity and human rights, and acknowledges the right to self-defined gender identity, allowing for changes to gender, image, or birth name on one's identity card and birth certificate. In contrast to many settings, including the majority of jurisdictions in the United States [24], this law allows for changes to gender identity without a requirement of psychiatric evaluation or judge approval. This law further supports the right to the full development of one's person in line with one's chosen gender identity, and ensures the right to appropriate transgender health services. According to official government sources, over 3000 transgender individuals have acquired new government issued identification since the implementation of the new law [25].

The Argentinean Gender Identity Law has potential to improve conditions for transgender individuals, since acknowledging the right to self-defined gender identity can be an important basis for inclusion of transgender individuals as citizens, and subsequent access to other legal, political and social rights. However, as with any public policy, its success ultimately relies on reaching the target population, especially those who are most vulnerable within an already marginalized group. Thus, identifying the barriers and facilitators to engagement with this law is critical to maximize its impact in addressing the unique needs of this vulnerable population. As indicated above, one of the essential components of this new law is the right to self-defined gender identity, including having access to identity documents that accurately reflect an individual's current name, gender identity, and gender expression. Therefore, the aim of this study was to explore potential individual and socio-structural factors associated with obtaining a new gender-congruent ID card among transwomen in Argentina.

## Materials and Methods

### Ethics statement

The study was approved by the institutional ethics committee of Fundación Huésped. All participants provided written informed consent. Participation was voluntary, and upon completing participants received a $100 ARS reimbursement (approximately $10 USD) for their time and effort.

### Participants and study design

Data for the present analysis were derived from a nationwide, semi-structured, cross-sectional survey conducted by Fundación Huésped and A.T.T.T.A. (Association of Transvestites, Transsexuals, and Transgender of Argentina), between June 2013 and December 2013.

Self-identified transgender individuals were recruited by outreach efforts coordinated by A.T.T.T.A., and occurred in 20 cities throughout Argentina, with outreach focusing in sex work venues and community-based organizations known to be frequented by transwomen. We first considered using snowball sampling, a commonly used method to recruit “hard-to-reach” and vulnerable populations [26]. However, after discussions with A.T.T.T.A. representatives, we realized that this strategy would result in the over representation of transwomen connected with advocacy organizations. Therefore, in order to maximize representativeness of the transgender population in Argentina, snowball sampling was combined with quota sampling [27]. Using statistics obtained from the national registration office (Registro Nacional de las Personas, RENAPER) and other national reports of socio-demographic characteristics of transgender individuals in Argentina, quotas were set for 6 age categories (14–19, 20–29, 30–39, 40–49, 50–59, and 60–65 years), 5 educational levels (no studies, incomplete primary education, complete primary education, incomplete high school education, complete high school education or greater) and the 6 Argentinean regions (Buenos Aires metropolitan area, the Pampas, Northeast, Northwest, Cuyo, and Patagonia).

After providing written informed consent, enrolled individuals completed an interviewer-administered questionnaire that captured information regarding socio-demographic characteristics, gender enhancement or transition procedures, self-reported HIV status, interactions with police, healthcare access, housing, education, work, and experiences of stigma and discrimination in these settings. The survey was conducted by trained transgender peers.

### Primary outcome and data analysis

For the current study, the primary outcome of interest was acquirement of a new gender-congruent ID card, defined as answering “yes” to the following question: “Have you obtained a new ID card since the enactment of the Gender Identity Law in May 2012?”

The following socio-demographic characteristics and social-structural factors were considered to be potentially associated with the outcome: age (greater than vs. less than median age), place of birth (Argentina vs. others), residency (Buenos Aires metropolitan area, the biggest urban center in Argentina, vs. others), current sexual partnership status (stable vs. others), peer support (defined as responding “yes” to the question: “are you a member of any transgender support group?”), having a job other than sex-work (yes vs. no), history of sex work (yes vs. no), having extended health insurance, defined as having either social security or private health coverage (yes vs. no), high school education level or higher (yes vs. no), housing status (stable vs. others), engagement in transition procedures (defined as responding “yes” to any of the following questions: “have you undergone any of the following procedures to appear more feminine: hormone treatment, aesthetic surgery, sex reassignment surgery, implants, industrial silicone injection?”), self-reported HIV status (positive vs. negative or unknown), internal stigma (defined as responding “yes” to any of the following questions: “have you ever feel any of the following emotions because of your transgender identity: ashamed, guilty, low self-esteem, feel that you should be punished?”), discrimination by healthcare workers (including physicians, psychologists, social workers, and other administrative staff), discrimination in educational settings (including institution directors, teachers/professors, classmates and other administrative staff), discrimination from social activities (including family, religious, and other social events), ever arrested (yes vs. no), and experiences of police or client violence (defined as answering “yes” to any of the following questions: “have you ever been exposed to any of the following situations due to your transgender identity: a policeman threatened you, a policeman beat, kicked or physically hurt you, a policeman forced you to have sex against your will, or client perpetrated violence, including physical, psychological and sexual violence?”).

Chi-square tests were performed to examine potential associations of each of these variables with acquiring a new ID. Variables found to be associated with the outcome at *p*<0.10 in bivariate analyses were included in a multiple logistic regression model. All differences were considered statistically significant at the two-tailed *p*-value <0.05 threshold. Analyses were performed using Stata/SE version 11.1 (Stata Corp, College Station, Texas).

## Results

Overall, 452 self-identified transwomen completed the survey and were included in this study. The majority of the participants were born in Argentina (90.0%), the median age was 31 years (interquartile range [IQR]: 25–37), and only 33.8% have completed high school. Three hundred and seventy eight participants (84.6%) reported a history of sex work (61.1% were currently engaged in sex work), and among those with previous HIV testing (n = 380), 27.4% being HIV-infected. As of December 2013, 260 (57.5%) reported that they had obtained new gender-congruent ID cards since the Gender Identity Law was passed in May 2012.

Results of bivariate analyses are displayed in Table 1. Factors positively and significantly associated with acquiring a new ID within the first 18 months of enactment of the Gender Identity Law were: previous experience of discrimination by healthcare workers, ever having engaged in transition procedures, having extended health insurance, and having a job other than sex work (all *p*<0.05). In contrast, foreign-born participants were less likely to have obtained a new ID (*p*<0.001).

**Table 1:** Bivariate analysis of factors associated with acquiring a new gender-congruent ID among transwomen in Argentina (n = 452).

Characteristic	Yes n (%) * n = 260	No n (%) * n = 192	Odds Ratio (95% CI)	*p -* value
**Age≥30 years**				
yes	141(54.2)	96 (50.0)	1.18 (0.82–1.72)	0.373
no	119 (45.8)	96 (50.0)		
**Peer support**				
yes	97 (38.3)	72 (39.1)	0.97 (0.66–1.43)	0.867
no	156 (61.7)	112 (60.9)		
**Stable partner**				
yes	92 (36.4)	75 (39.5)	0.88 (0.59–1.29)	0.504
no	161 (63.6)	115 (60.5)		
**Extended health insurance**				
yes	59 (23.6)	22 (11.6)	2.34 (1.38–3.99)	0.001
no	191 (76.4)	167 (88.4)		
**Transition procedures**				
yes	240 (92.7)	164 (85.4)	2.16 (1.17–3.99)	0.013
no	19 (7.3)	28 (14.6)		
**Ever arrested**				
yes	208 (80.0)	146 (77.7)	1.15 (0.73–1.82)	0.548
no	52 (20.0)	42 (22.3)		
**Discrimination by healthcare workers**				
yes	184 (70.8)	118 (61.5)	1.52 (1.02–2.25)	0.038
no	76 (29.2)	74 (38.5)		
**Discrimination in educational settings**				
yes	207 (79.6)	150 (78.1)	1.09 (0.69–1.73)	0.701
no	53 (20.4)	42 (21.9)		
**Discrimination from social activities**				
yes	135 (51.9)	117 (60.9)	0.69 (0.47–1.01)	0.056
no	125 (48.1)	75 (39.1)		
**Sex work ever**				
yes	216 (83.4)	162 (86.2)	0.81 (0.48–1.37)	0.423
no	43 (16.6)	26 (13.8)		
**Stable housing**				
yes	212 (81.5)	143 (74.5)	1.51 (0.96–2.38)	0.071
no	48 (18.5)	49 (25.5)		
**Client/police violence**				
yes	107 (41.2)	129 (67.2)	0.70 (0.47–1.03)	0.070
no	153 (58.8)	63 (32.8)		
**High school education or greater**				
yes	87 (33.6)	65 (34.0)	–0.98 (0.661.46)	0.922
no	172 (66.4)	126 (66.0)		
**Self-reported HIV status**				
positive	56 (25.2)	48 (30.4)	–0.77 (0.491.22)	0.267
negative	166 (74.8)	110 (69.6)		
**Internal stigma**				
yes	132 (50.8)	113 (58.9)	–0.72 (0.491.05)	0.088
no	128 (49.2)	79 (41.1)		
**Employed (other than sex work)**				
yes	79 (30.4)	29 (15.1)	–2.45 (1.533.95)	<0.001
no	181 (69.6)	163 (84.9)		
**Residency in Buenos Aires**				
yes	86 (33.1)	54 (28.1)	–1.26 (0.841.90)	0.260
no	174 (67.0)	138 (71.9)		
**Foreign born**				
yes	9 (3.5)	36 (18.7)	–0.16 (0.070.33)	<0.001
no	251 (96.5)	156 (81.3)		

* Totals may differ due to non-response on some questions.

CI: confidence interval.

As shown in Table 2[]%[]– = %– = %– = %–, previous experiences of discrimination by healthcare workers (adjusted odds ratios aOR 2.01, 95 confidence intervals CI: 1.273.20), having engaged in transition procedures (aOR3.06, 95 CI: 1.575.93), and having a job other than sex work (aOR1.81, 95 CI: 1.063.10) remained positively associated with acquisition of a new ID card in the multivariate model. Additionally, foreign-born status remained as an independent and strong negative correlate of new ID acquisition (aOR0.14, 95 CI: 0.060.33). Importantly, educational level, housing conditions, self-reported HIV status and history of sex work were not associated with the outcome.

**Table 2:** Multivariate logistic regression of factors associated with acquiring a new gender-congruent ID among transwomen in Argentina (n = 452).

Variable	Adjusted Odds Ratio (AOR)	%95 Confidence Interval (CI)	*p -* value
**Discrimination from social activities**			
(yes vs. no)	0.75	–(0.471.18)	0.217
**Discrimination by healthcare workers**			
(yes vs. no)	2.02	–(1.273.20)	0.003
**Stable housing**			
(yes vs. no)	1.36	–(0.822.28)	0.236
**Client/police violence**			
(yes vs. no)	0.86	–(0.541.36)	0.521
**Internal stigma**			
(yes vs. no)	0.85	–(0.551.30)	0.444
**Employed (other than sex work)**			
(yes vs. no)	1.81	–(1.063.10)	0.029
**Foreign born**			
(yes vs. no)	0.14	–(0.060.33)	<0.001
**Transition procedures**			
(yes vs. no)	3.06	–(1.585.93)	0.001
**Extended health insurance**			
(yes vs. no)	1.41	–(0.782.55)	0.251

CI: confidence interval.

## Discussion

In this study, more than half of the transwomen surveyed had obtained a new gender-congruent ID card within 18 months of enactment of the Gender Identity Law. This finding is encouraging, since having a gender-congruent ID is the first step for inclusion of transgender individuals as legal and legitimate citizens, and a pre-requisite for access to welfare and social programs, employability and other civil and political rights. Somewhat concerning, however, is the finding that while some sub-groups within this already marginalized population, including transwomen with low level education, unstable housing conditions, and HIV infection, have taken advantage of this new policy initiative, these characteristics were not independently associated with having accessed new ID cards. On the contrary, acquisition of a new ID card was positively associated with experiences of discrimination in healthcare settings, transition procedures, employment, and Argentinean nationality, suggesting that access to and engagement with this law has not been completely homogenous among this population.

Multiple studies have documented high rates of discrimination against transwomen within healthcare facilities, which can result in transwomen postponing or avoiding healthcare due to previous experiences of discrimination and disrespect [4], [8], [28], [29]í. A recently completed but as of yet unpublished qualitative study among transwomen in Argentina has shown that obtaining a new ID card has for many served to increase access to health services, in particular access to gender enhancement procedures and HIV care (Arstegui, unpublished data). Altogether, these findings suggest that transwomen may perceive the opportunity to update their ID according to their gender identity as a resource to decrease the social stigma they face, and, particularly within the health system, as a tool to access timely and appropriate healthcare.

“”'Body modification procedures, including hormones, surgeries or injection of industrial silicones (referred to by members of the population as airplane oil), are a potentially important part of transwomens identity affirmation [30], [31]. However, due to the barriers in accessing appropriate healthcare, transition procedures usually occur outside the health system, with the consequent risk associated with the use of non-medical products in non-sterile environments [32], [33]''. The finding that engagement in transition procedures was positively associated with obtaining a new ID card in the multivariate analysis might be a reflection of empowerment or commitment related to ones transgender identity, or ultimately as a means to finally attain identity recognition that is congruent with ones gender and body image. Additionally, transwomen who have initiated such transitions without medical supervision may be seeking another benefit of the law, such as the right to comprehensive transgender health services, including proper medical conditions for gender enhancement procedures, with regular monitoring of their health. Further research is needed to better understand perceptions of transition procedures in this population, particularly in the context of new legal protections provided by federal Argentinian laws.

Interestingly, transwomen who were currently employed in a job other than sex work were more likely to have updated their ID card. Although one could argue that transwomen who have been denied a job due their transgender status would be most likely to try to change their ID, it might also be possible that employment functions as a proxy of higher levels of empowerment, education, and social and family support among this population. Accordingly, employed transwomen may be better prepared to navigate through the complexities of the public bureaucracy to successfully obtain a new ID.

Finally, although only one tenth of the participants reported non-Argentinean nationality at birth (all of them from other South-American countries), foreign born status was a strong negative correlate of new ID acquisition. This finding raises serious public health concern, as migrant populations face additional challenges due to their migration status, such as stigmatization, social isolation, health risks, and language or cultural barriers, which can also negatively impact their health outcomes [34], [35]. These risks are further exacerbated in undocumented immigrants [36], [37]. Future research is needed to better understand how intersecting, marginalized identities shape health outcomes and living conditions among transwomen, as well as potential interventions for reaching these communities [38]–[40].

The present study has several limitations. First, as there are no official registries of transwomen in Argentina, our sample was not randomly selected, and therefore we cannot assume that our results are generalizable to all transwomen in Argentina. We tried to mitigate this potential source of bias by recruiting a large sample, and by combining snowball and quota sampling to ensure the recruitment of transwomen from different age groups, educational levels and regions. Second, the self-reported data may have been affected by socially desirable responding or recall bias. We employed and trained transgender peers to administer the surveys in an attempt to mitigate this bias. Third, the use of cross-sectional data does not allow for causal inference. Therefore, the associations presented herein should be interpreted with caution, and longitudinal studies are recommended to further examine the observed relationships. Fourth, we did not have specific information about the country of birth of the foreign participants. Therefore, we were unable to determine whether the relationship with the outcome varied among different non-Argentinean nationalities. Lastly, although we performed multivariate analysis to adjust for known relevant confounders, there may be unmeasured factors that may confound the relationship between exposures and outcomes.

In summary, we found that more than half of transwomen in our sample have acquired a new gender-congruent ID card within the first 18 months of enactment of the Gender Identity Law. Although this finding is encouraging, access to and uptake of this right has not been homogenous. In particular, our analysis suggests that the most empowered transwomen (e.g., transwomen with stable jobs or who have previously undergone transition procedures) may have been among the first to take advantage of this right. However, further research is needed to confirm this interpretation. As has been previously shown, gender-incongruent identification presents barriers to health care, employment, housing and education, as well as exposes transwomen to violence [4]. Therefore, further research is needed to better understand how the intersection of micro-, meso- and macro-level factors shape engagement with the Gender Identity Law, in order to ensure that benefits of this founding policy reach all transwomen living in Argentina.
